# Exploring the Interplay of Motivation, Self-Efficacy, Critical Thinking, and Self-Regulation in Predicting Academic Achievement Among University Students

**DOI:** 10.12688/f1000research.161821.2

**Published:** 2025-07-07

**Authors:** Georgia Stavropoulou, Athena Daniilidou, Katerina Nerantzaki

**Affiliations:** 1 Aristoteleio Panepistemio Thessalonikes Philosophike Schole, Thessaloniki, Makedonia Thraki, Greece; 2 Panepistemio Makedonias, Thessaloniki, Makedonia Thraki, Greece

**Keywords:** critical thinking, motivation, self-efficacy, self-regulation

## Abstract

**Background:**

Motivational variables are of critical importance concerning students’ performance. The objective of the present study was to investigate the interrelationships between extrinsic and intrinsic motivation, self-efficacy, self-regulation, critical thinking, and academic performance among university students.

**Methods:**

The participants were 250 students enrolled in university programs in education and psychology. The research instrument was a self-report questionnaire designed to assess intrinsic and extrinsic motivation, critical thinking, self-regulation, and academic achievement among university students. A path model analysis was employed to identify the relationships among the investigated variables.

**Results:**

The results demonstrated that self-efficacy was predicted by intrinsic and extrinsic motivation, critical thinking was predicted by self-efficacy, and self-regulation was predicted by self-efficacy and critical thinking, thereby underscoring the pivotal role of self-efficacy. The findings indicate that academic achievement is predicted by critical thinking, and self-regulation, thereby underscoring the pivotal role of these variables in academic contexts.

**Conclusions:**

The contributions of the present research are twofold, both theoretical and practical. On the one hand, the findings offer a more nuanced understanding of the interconnections between motivation, self-efficacy, critical thinking, and self-regulation. On the other hand, they provide valuable insights for developing educational strategies that enhance academic achievement by fostering these key factors.

## Introduction

A substantial body of education research has consistently demonstrated that prior academic performance is the most reliable predictor of future success ( Alyahyan & Düştegör, 2020; Zeegers, 2004). However, achieving academic success in higher education is a complex outcome influenced by a constellation of motivational and cognitive factors. While numerous studies have explored individual predictors such as prior academic performance, personality traits, or emotional engagement (e.g., Collie et al., 2017; Greene & Yu, 2016; Komarraju et al., 2011), less attention has been given to the integrated interplay among key constructs like motivation, self-efficacy, critical thinking, and self-regulation. These factors are especially critical in university settings, where students are expected to manage autonomy, engage in abstract reasoning, and adapt to increasingly self-directed learning environments ( Girelli et al., 2018).

In this context the difficulties encountered by university students in attaining academic performance have long been a matter of grave concern for institutions of higher learning. In the context of higher education, academic success can be defined as the ability of students to successfully complete a given semester, which is a prerequisite for promotion to the subsequent academic year and the completion of the university program. A substantial body of education research has consistently demonstrated that prior academic performance is the most reliable predictor of future success (e.g., Alyahyan & Düştegör, 2020; McKenzie & Schweitzer, 2001; Zeegers, 2004). Moreover, studies in educational psychology have examined the relationship between various factors and academic achievement. These factors including adaptability, behavioral engagement (e.g., Collie et al., 2017), personality traits ( Komarraju et al., 2011; Laidra et al., 2007), self-efficacy ( Hwang et al., 2016; Liem et al., 2008), emotional engagement ( Gonida et al., 2009), self-regulation (e.g., Meece & Painter, 2012) and critical thinking ( Greene & Yu, 2016; Halpern, 2013).

Motivation for academic success is inextricably linked to behaviors that facilitate effective learning and achievement (e.g., Hulleman et al., 2017). It comprises a robust motivation to complete tasks efficiently within a given context and to evaluate performance promptly. While extensive research has been conducted in elementary and secondary education regarding motivational variables and their impact on student performance (e.g., Stavropoulou et al., 2023, 2025; Stavropoulou & Stamovlasis, 2024, 2025; Friedel et al., 2007; Stamovlasis & Gonida, 2018; Wormington & Linnenbrink-Garcia, 2017), a notable gap exists in exploring these factors within the context of higher education. Specifically, research focusing on the interconnectedness of motivation, self-efficacy, self-regulation, and critical thinking in university students remains limited. This is a significant oversight, as these cognitive and motivational constructs are likely to play an equally, if not more, critical role in higher education, where it is anticipated that students will engage in complex learning tasks, develop autonomy, and apply critical thinking skills in more advanced and diverse academic settings (e.g., Washer, 2007). Despite the recognized importance of motivation, self-efficacy, and self-regulation in learning, the absence of comprehensive studies examining their specific impact on other thinking skills such as critical thinking in a university environment represents a clear research gap that needs to be addressed. Understanding how these factors interact in a university context could provide valuable insights into improving student engagement, performance, and academic success at the postsecondary level, and help educators design interventions that foster these key skills.

In light of these considerations, the current study aimed to address this gap by examining the interrelationships between various motivational variables and their influence on academic achievement within a university setting. The study aims to provide a better understanding of how motivation interacts with important cognitive factors that contribute to student success.

## Literature review

### Defining motivation, self-efficacy, critical thinking, and self-regulation

***Motivation***

Motivation is a complex aspect of human psychology and behavior that influences several factors, including the allocation of time, the selection of learning tasks, the level of effort invested in a task, the thoughts and feelings associated with the task, the duration of persistence in completing the task, and the ability to overcome challenges encountered during the learning process ( Ryan & Deci, 2000b). Moreover, motivation can be defined as a process that originates from either a psychological or physiological need. This need then initiates a behavior or drive directed toward a specific goal or incentive ( Ryan & Deci, 2017, 2020). It is evident that learners ascribe divergent meanings and attitudes to the academic environment, which in turn inform their actions and channel their energy in a variety of directions. These invigorating and instructive influences are designated as motivation or the motivation to learn. Motivation has been identified as a crucial factor in the success of the teaching-learning process. As the term suggests, motivation is defined as the driving force behind all human action and behavior ( Ryan & Deci, 2017, 2020).

The Self-Determination Theory (SDT) framework proposes that learners are motivated by both intrinsic and extrinsic factors. Intrinsic motivation arises from within the individual and can be influenced by emotional, spiritual, biological, or social factors. In this form of motivation, no external rewards are sought; instead, activities are pursued for their inherent value and personal satisfaction ( Gagné & Deci, 2005; Ryan & Deci, 2000a, 2017, 2020). It is often characterized by curiosity, interest, and a desire to overcome challenges, which can be influenced by both intrinsic and extrinsic factors ( Niemiec & Ryan, 2009). Intrinsic motivation refers to engaging in activities for their own sake, independent of external rewards, with the reward residing in the activity itself. This type of motivation represents a fundamental psychological drive for exploration, engagement, and mastery, key components of human development and lifelong learning ( Ryan & Deci, 2017, 2020).

Moreover, intrinsic motivators, apart from a profound interest in the subject matter, are characterized by a perception of its relevance to life and a sense of accomplishment in mastering it ( Cavallo et al., 2003; Matt & Dale, 2002). Such individuals engage deeply in mental and physical activities, maintaining a high level of focus and a clear understanding of their objectives. Such individuals are self-critical, capable of reflecting on their actions realistically, and typically approach learning with a relaxed attitude, not fearing failure. Those intrinsically motivated to learn seek to expand their knowledge base, deriving positive emotions from the process and often demonstrating a superior grasp of the subject matter. Intrinsic motivation is fundamental to most human learning processes and has a notably positive impact within formal education. The findings showed that intrinsic motivation to know and to accomplish were moderately strong predictors of positive student outcomes. Intrinsic motivation to experience stimulation was also positively linked to beneficial outcomes ( Howard et al., 2020). For example, Taylor et al. (2014) conducted a meta-analysis demonstrating the important role intrinsic motivation plays in enhancing academic performance. Likewise, Froiland and Worrell (2016) showed that intrinsic motivation increases student engagement, which subsequently contributes to improved GPA scores, while it has been also proved that it is associated with engagement ( Kotera et al., 2023).

Conversely, extrinsic motivation has also been demonstrated to influence the learning process. Extrinsic motivation is defined as any stimulus that originates from external sources, potentially involving social cognition or operant conditioning, which prompts the student to engage in the learning process (see Chow & Yong, 2013; Hewett & Conway, 2016). In contrast to intrinsic motivation, which is autonomous and internalized, extrinsic motivation is dependent on external incentives, pressures, or consequences ( Ryan & Deci, 2020). For instance, a student driven by the aspiration to attain a commendable grade or to circumvent failure is regarded as being extrinsically motivated. In competitive settings, students frequently participate in activities with the objective of outperforming others rather than for the sake of pure enjoyment, which also reflects extrinsic motivation ( Featherstone & Habgood, 2019). Consequently, the promotion of competition or collaboration among students is frequently regarded as a strategy to enhance extrinsic motivation in learning environments.

Intrinsic motivation is often more influential than extrinsic motivation because it originates from within the learner and is unaffected by external factors. Intrinsic motivation can also be more enduring and self-perpetuating than extrinsic motivation, which lasts less because removing rewards or punishments results in a loss of motivation among students ( Matt & Dale, 2002). Extrinsic rewards may negatively influence intrinsic motivation. Individuals motivated extrinsically depend entirely on rewards and desirable outcomes for their drive ( Lei, 2010). Consequently, students with external motivation are likelier to perform at a lower academic level than intrinsically motivated students ( Lei, 2010). Previous research has demonstrated that learners with stronger motivations for learning tend to achieve better learning outcomes ( Giesbers et al., 2013; Sansone et al., 2012).

Motivation has been widely acknowledged as a pivotal factor influencing academic behavior, with many theories striving to elucidate and explain the motivational processes that culminate in academic outcomes, particularly achievement. It is of paramount importance to motivate learners in order to ensure effective curriculum implementation, as motivation has a significant impact on the dynamics of teaching and learning ( Harackiewicz et al., 2002; Pintrich, 2004; Urdan & Kaplan, 2020). The extent to which the learning process is effective is contingent upon the level of motivation exhibited by the learners, which propels them toward attaining their educational goals. It is imperative to recognize that fostering motivation is fundamental to effective teaching ( Hulleman et al., 2017; Senko & Dawson, 2017; Stavropoulou et al., 2023; Urdan & Kaplan, 2020).

A crucial aspect of understanding motivation is also the concept of self-efficacy.

***Self-efficacy***

The significance of self-efficacy beliefs in shaping human behavior is well-recognized within the framework of social cognitive theory ( Bandura, 1997, 2006). The motivational influence of perceived self-efficacy and its ability to predict performance or interest in different areas, including academics, has been well established from a substantial body of empirical evidence, along with numerous meta-analytic studies ( Bandura & Locke, 2003; García-Martín & García-Sánchez 2018; Panadero et al., 2017). Bandura (1997), Schunk and Pajares (2005) defined perceived self-efficacy as an individual’s belief in their ability to learn or perform tasks at specific levels. Self-efficacy beliefs influence people’s decisions and actions, determining the amount of effort they invest in an activity and their persistence when facing challenges and it has been proved to be one of the most influential factors that predict academic achievement ( Alhadabi & Karpinski, 2020; Celik, 2022; Sheu et al., 2018). Additionally, they influence how individuals perceive themselves, pivotal in forming the self-concept ( Bong & Skaalvik, 2003) and assuming individuals possess sufficient skills and other motivational factors are favorable, self-efficacy plays a crucial role in driving strong motivational outcomes ( Schunk & DiBenedetto, 2020). Various sources can influence individuals’ beliefs regarding their efficacy. Mastery experiences are the most efficacious methods for developing a robust sense of efficacy, as successes bolster confidence in one’s abilities. Conversely, failures can erode this belief, mainly if they occur before a robust sense of efficacy has been firmly established ( Bandura & Wessels, 1997; Schunk & DiBenedetto, 2020).

Nevertheless, self-efficacy beliefs may be accurate or inaccurate reflections of an individual’s competence. Individuals may engage in self-enhancing or self-limiting cognitive processes when these beliefs are demonstrably inaccurate (e.g., Bouffard et al., 2003; Schunk & DiBenedetto, 2020). In an educational context, students may either overestimate or underestimate their self-efficacy relative to their actual academic abilities. In other words, their self-evaluation of their academic skills can be affected by either a positive or negative bias, leading to an inflated sense of their abilities or a lack of confidence in their competencies ( Bouffard et al., 2003; Schunk & DiBenedetto, 2020). Motivation and self-efficacy have been demonstrated to relate to other abilities that engage in the learning process, including critical thinking.

***Critical thinking***

The capacity for critical thinking is also a crucial component of the academic process. Critical thinking involves applying previous knowledge to unfamiliar situations, challenges, decisions, or criteria for excellence. Critical thinking is elucidated as the application of cognitive skills or strategies with the objective of achieving long-term desired outcomes through goal-directed, “high-level” cognitive processes such as judgment, analysis, and synthesis of information ( Halpern, 2013). In order to assess the quality of an argument effectively, it is necessary to evaluate a number of factors, including the logic employed, the strength of the evidence presented, the credibility of the sources used, the identity of the person making the argument, and the potential for counterarguments to be made ( Greene & Yu, 2016). Specifically, it involves analyzing, assessing, and solving problems ( Rodzalan & Saat, 2015; Jatmiko et al., 2018). A critical thinking process is systematic and structured, whereby conclusions are formulated and assessed based on evidence, assumptions, and justifiable logic ( Sari et al., 2019). Critical thinking is an essential intellectual process, without which the understanding of scientific concepts would be severely impeded. It enables students to analyze their thoughts effectively, make informed choices, and conclude intelligently.

Developing critical thinking skills allows students to navigate natural and social environments with practical and effective expertise. The advancement of critical thinking skills necessitates a rational and systematic approach. The process entails continuously observing and analyzing similarities, differences, and causal relationships ( Florea & Hurjui, 2015). It is paramount for students to develop critical thinking skills, as this enables them to swiftly discern reliable information, focus on practical learning and become attuned to real-world situations.

Consequently, critical thinking is paramount in addressing traditional learning challenges, such as knowledge transfer and applying problem-solving skills in unfamiliar contexts. In many countries, educational systems have increasingly emphasized creativity, critical thinking, problem-solving, and decision-making as essential in advancing 21st-century education ( Wechsler et al., 2018). Therefore, this shift, which facilitates the growth of critical thinking skills, has transformed the concept of traditional education and advanced it towards a more modern approach. The development of critical thinking is contingent upon interaction with other individuals engaged in the same process of critical thinking. In order to make logical decisions and select the optimal course of action, it is essential to analyze data and apply critical thinking skills ( Greene & Yu, 2016). It is of interest to examine the association between critical thinking and self-regulation. Similarly, the capacity for self-regulation is similarly of great consequence in academic contexts.

***Self-regulation***

Specifically, self-regulation is the process by which students generate their thoughts and behaviors in a structured way in order to achieve their learning goals. This type of learning involves goal-directed actions that students initiate, adapt, and maintain. Examples include paying attention in class, processing information and relating new knowledge to what they already know, and creating compelling social interactions and productive work environments ( Pintrich & De Groot, 1990). Self-regulated learning aligns with the view that students are not passive receivers of information but rather active participants in setting and pursuing their learning goals, taking responsibility for their own achievement ( Meece & Painter, 2012; Pintrich & De Groot, 1990). Self-regulated behavior involves making choices between different courses of action, often by delaying immediate gratification in favor of a greater reward in the future. Self-regulated learning requires that students understand the task’s requirements, recognize their abilities, and use effective strategies to complete the task. Notably, as students enter adolescence, they must develop self-regulated academic competence that enables them to systematically adjust their strategies in response to changes in various conditions ( Bandura, 1997).

The ability to self-regulate one’s thoughts and actions is essential for effective learning and academic achievement (e.g., Corno & Mandinach, 1983). Self-regulation includes multiple components. To begin with, self-regulated learning involves students applying metacognitive strategies to plan, observe, and adapt their thought processes (e.g., Zimmerman & Pons, 1988). A key aspect of self-regulated learning is students’ ability to manage and regulate their effort when working on academic tasks in the classroom. For example, skilled students who persevere through challenging tasks or block out distractions can maintain cognitive focus, improving performance. Moreover, a third important element of self-regulated learning, highlighted by some researchers, is the cognitive strategies students employ to understand, retain, and process information ( Corno & Mandinach, 1983; Zimmerman & Pons, 1988).

Self-regulated learning involves self-directed processes and beliefs that allow learners to convert their cognitive abilities into academic skills. It is understood as a proactive strategy that students employ to develop academic skills, including setting goals, choosing and applying strategies, and monitoring their effectiveness, rather than being a passive response to external forces. Although self-regulated learning is especially important in self-directed activities like discovery learning, self-selected reading, or online research, it is also crucial in social learning environments, such as seeking assistance from peers, parents, or teachers. The key factor is whether a learner shows personal initiative, perseverance, and adaptability. These proactive qualities emerge from positive motivational beliefs, emotions, and metacognitive strategies.

***Relationship among motivation, self-efficacy, critical thinking, and self-regulation***

*Self-efficacy with motivation, critical thinking, and self-regulation*

All of the above variables play an essential role in the academic environment. Self-efficacy is related to motivation ( Pintrich & De Groot, 1990) and especially to intrinsic motivation ( Bandura, 1997; Greene et al., 2004; Walker et al., 2006). Moreover, when someone has high self-efficacy and high motivation, there is better performance ( McGeown et al., 2012). Conversely, there is poorer performance when there is low self-efficacy ( Wolters et al., 2014). Self-efficacy is arguably one of the most significant predictors of performance. It not only influences the level of effort and persistence an individual invests in challenges but also significantly affects their motivation and approach to overcoming obstacles and attaining success (e.g., Liem et al., 2008; Pajares, 2003).

Research on the relationship between self-efficacy and critical thinking is still in infancy. Recent efforts have been made to explore and facilitate the establishment between these two concepts. Critical thinking is also related to self-efficacy. Specifically, self-efficacy may influence critical thinking because people with high levels of self-efficacy tend to be more diligent in their learning and less inferior in any situation ( Ng et al., 2016). Phan (2007) found that academic self-efficacy positively influenced comprehension and reflection but not critical thinking ( Leung & Kember, 2003). Students with high self-efficacy are more likely to engage in learning that challenges their assumptions, beliefs, and perceptions.

In studies related to self-regulation, only Panadero and colleagues ( Panadero et al., 2012, 2013) appear to have examined the effects of rubric, script, and exemplar feedback on performance, self-efficacy, and self-regulation. Their findings indicated that feedback did not significantly affect self-regulation, and only process-oriented feedback led to increased self-efficacy. Some research has examined the relationship between students’ views of assessment and self-regulation. Students who view assessment as a tool for improvement tend to exhibit adaptive self-regulatory behaviors, such as higher achievement, more effort on tests, and better attendance on voluntary test days. In contrast, those who view assessment as something to be ignored often display maladaptive behaviors. Feedback related to assess performance enhances academic self-efficacy and self-regulatory processes, which in turn affect future learning and achievement ( Brown, 2011). In addition, higher academic self-efficacy is positively associated with effective self-regulated learning ( Richardson et al., 2012). Academic self-efficacy and self-regulated learning are related to beliefs about competence and control ( Schunk & Zimmerman, 2006), contributing to higher achievement. Furthermore, students with high self-efficacy in reading and writing are more likely to use deep or strategic study approaches. In contrast, those with low self-efficacy tend to use surface approaches. Notably, changes in student study methods are related to their self-efficacy beliefs. Students with lower self-efficacy show a decrease in deep study approaches and an increase in surface approaches over time.

*Critical thinking and motivation*

Engaging in a task for intrinsic motivations such as interest, mastery, or challenge is associated with “deeper” information processing ( Cavallo et al., 2003; Matt & Dale, 2002). In contrast, engagement for extrinsic reasons, such as demonstrating one’s ability, earning good grades, or outperforming others, is associated with more superficial processing (e.g., Chow & Yong, 2013; Niemiec & Ryan, 2009; Ryan & Deci, 2000a). Research has highlighted the vital role of motivation in students’ cognitive engagement. Critical thinking represents higher-order cognitive engagement. It can be reasonably deduced that students who utilize deep learning strategies will exhibit a higher degree of critical thinking than those who rely on surface-level strategies. Critical thinking is shaped by student motivation, learning strategies, and classroom dynamics ( Ames & Archer, 1988; Nolen, 1988). Students who work together in small peer groups tend to show greater cognitive engagement.

In addition, motivation and the authenticity of the problems posed are critical to fostering critical thinking. Motivated students are more diligent in their search for the correct solution, which increases their focus and enables them to filter out irrelevant information and focus only on what is necessary. When faced with authentic problems requiring innovative solutions, students are motivated to work beyond the classroom. They engage in hypothesis generation, analysis, synthesis, evaluation and the real-time presentation of their findings. However, critical thinking skills are usually low in learning environments ( Din, 2020).

*Self-regulation with motivation and critical thinking*

The implementation of self-regulation strategies has been demonstrated to be a significant predictor of academic performance, as well as a contributing factor in the evaluation of proactive learning efforts by educators ( Zimmerman & Martinez-Pons, 1988). The variables that comprise academic performance include internal locus of control, intrinsic motivation, and perceived self-efficacy. The self-regulation variable demonstrates a robust correlation with motivation, indicating that elevated levels of self-regulation are associated with heightened motivation ( Zimmerman, 2008). This also supports the idea that self-regulation moderates the relationship between achievement and motivation ( Zimmerman, 2008). The scores related to learning outcomes and performance strongly correlate with all aspects of self-regulation and motivation except for external regulation. This suggests that students are not driven to learn by external pressures or to satisfy essential adults, such as parents or teachers ( Daniela, 2015). Daniela (2015) also highlighted students’ recognition of their responsibility for personal development. Greater self-confidence fosters internal motivation, enabling students to regulate their internal processes, validate their results against appropriate standards, and exceed their academic performance. Thus, academic performance improves when individuals know their goals, regulate and control their impulses, follow the rules, prefer careful planning, and demonstrate perseverance to succeed.

Consequently, when students align with internal values, follow their satisfaction standards, and view learning as necessary, they achieve higher academic success. Positive beliefs about the academic institution, the performance of activities, and the pursuit of goals are associated with high academic achievement. Furthermore, confidence in their ability to mobilize cognitive resources and motivation necessary for task completion is strongly correlated with high academic achievement ( Daniela, 2015). Self-regulated learning competence significantly impacts students’ academic performance, making it one of the most important transferable skills that schools should prioritize in their curriculum. Firstly, it enhances motivation and facilitates student autonomy in the learning process. Secondly, it indirectly encourages positive behavioral changes and improves overall academic performance ( Daniela, 2015).

Additionally, there is a dynamic interaction between self-regulatory skills and the capacity for critical thinking ( Phan, 2010). Phan (2010) posited that critical thinking, as a cognitive practice, serves to bolster self-regulation in the context of learning and teaching. Moreover, he posited that the complex interplay between these elements facilitates individual growth and development. Zimmerman (1990) argued that skills related to evaluation and reflective thinking are vital components of self-regulation. According to Zimmerman (1990), self-regulated students actively engage in their learning process through motivational, behavioral, and metacognitive efforts. These students demonstrate persistence, dedication in their studies, and high self-efficacy and intrinsic interest levels. In their metacognitive processes, self-regulated learners engage in goal setting, progress monitoring, and learning evaluation. Such an approach enables learners to develop self-awareness and to make informed decisions regarding their learning methodology ( Zimmerman, 1990). The capacity for self-awareness and self-evaluation are inextricably linked to individuals’ abilities to reason and reflect, which in turn constitute aspects of their critical thinking abilities. Critical thinking represents an advanced form of reflective thinking, entailing a more profound understanding of the underlying factors that shape our perceptions and influence our emotions and actions ( Phan, 2008). Self-efficacy is a critical determinant of the utilization of deep processing strategies ( Fenollar et al., 2007), and is associated with achievement goals, deep processing strategies, critical thinking ( Phan, 2007), and academic performance. Furthermore, evidence indicates that self-efficacy plays a significant role in influencing academic performance. Self-regulation plays a crucial role in enhancing critical thinking because it involves deliberate reflection, evaluation, and adjustment of one’s thinking strategies during problem-solving and decision-making. Research shows that students with strong self-regulatory skills tend to engage more deeply with learning materials, exhibit greater persistence, and apply higher-order thinking skills, all of which are essential components of critical thinking ( Ayhan & Payan, 2023; Tunçeli et al., 2022). Thus, self-regulation acts as a foundational process that supports the development and application of critical thinking in academic contexts.

The motivation exhibited by students is generally correlated with their utilization of specific self-regulatory processes ( Schunk & Zimmerman, 2007). For instance, Schmitz and Wiese (2006) reported significant gains from self-regulated across various motivational measures, including intrinsic motivation for studying, self-efficacy, effort, attention, self-motivation, managing distractions, and reducing procrastination. Motivational orientation and self-efficacy beliefs ( Bandura, 1997) have been recognized as individual difference factors linked to students’ study approaches ( Entwistle et al., 2000), academic performance ( Bong, 2001; Lane et al., 2004; Richardson et al., 2012), and self-regulated learning ( Daniela, 2015; Meece & Painter, 2012; Pintrich & De Groot, 1990; Schunk & Zimmerman, 1997). Recent research in the field of education has focused on four vital theoretical factors: (1) achievement goals, (2) self-efficacy, (3) critical thinking, and (4) study processing strategies. These motivational factors have been identified as a significant predictor and mediator of academic achievement outcomes ( Bandura, 1997; Elliot et al., 1999).

***Aim and research hypotheses***

A growing body of research has highlighted the complex relationship between motivational factors and academic performance. Self-efficacy, in particular, has emerged as a key predictor of various educational behaviors and cognitive processes ( Bandura, 1997; Greene et al., 2004; Walker et al., 2006). Specifically, a strong correlation has been observed between self-efficacy and motivation, particularly intrinsic motivation. This suggests that students who believe in their academic abilities are more likely to be self-motivated and committed to learning. Consequently, both extrinsic and intrinsic motivation are expected to predict students’ self-efficacy. Furthermore, a substantial body of research has investigated the relationship between motivational factors and academic performance in higher education (see Agustina et al., 2021; Stavropoulou et al., 2024; Pestana et al., 2023), indicating the association between motivation and academic performance.

Although extensive research has examined the individual roles of motivation, self-efficacy and critical thinking in academic contexts, fewer studies have investigated their interdependent effects using an integrative model, especially in diverse cultural and educational settings. Although motivation is widely recognized as a key factor in student performance, the interaction between different types of motivation and cognitive and self-regulatory mechanisms in influencing academic achievement remains unclear. Moreover, much of the existing literature tends to consider motivation, self-efficacy, critical thinking and self-regulation separately, overlooking the dynamic relationships between them.

Another key gap lies in the limited empirical attention given to how self-efficacy functions as a mediating construct linking motivational factors to higher-order thinking and self-regulated learning strategies. High self-efficacy has been associated with deeper engagement, lower academic anxiety, and more frequent use of deep processing strategies ( Fenollar et al., 2007; Phan, 2007), which are essential for critical thinking and academic performance. Yet, the mechanisms by which motivation translates into academic success through self-efficacy and critical thinking remain insufficiently theorized and tested. Moreover, although motivation is often emphasized as the most crucial factor influencing student learning outcomes, the justification for prioritizing motivation over other variables in academic research is often underdeveloped. In the current study, motivation is foregrounded not only because of its predictive power but also due to its foundational role in activating and sustaining the cognitive and metacognitive strategies that underpin learning success. Motivation is the catalyst that initiates and directs effort, making it the logical starting point for exploring the psychological pathways that lead to academic achievement.

In response to these gaps, the present study aims to contribute to the literature by proposing and empirically testing a path model that integrates intrinsic and extrinsic motivation, self-efficacy, critical thinking, self-regulation, and academic achievement. This approach enables a more holistic understanding of the psychological mechanisms underpinning student performance in higher education. Importantly, the study is situated within a specific cultural and institutional context where such integrative models have not been extensively tested, thereby adding contextual depth to the existing body of knowledge. Based on the preceding discussion, four interrelated hypotheses were proposed:

- Intrinsic and extrinsic motivation would positively predict self-efficacy (Hypothesis 1)
- Self-efficacy would serve as a predictor of critical thinking (Hypothesis 2)
- Critical thinking and self-efficacy would positively predict self-regulation (Hypothesis 3)
- Critical thinking, and self-regulation would positively predict achievement (Hypothesis 4)

Hypotheses are grounded in established theory, considering the relevant literature and the specific context of the current study. The proposed design and hypotheses offer a fresh perspective on academic achievement, and the anticipated findings are expected to further contribute to the theoretical foundations of the field.

## Method

### Participants

The current study’s participants were 250 undergraduate students enrolled in Education and Psychology study programs at Greek universities, specifically at the Aristotle University of Thessaloniki, the University of Western Macedonia in Florina, and the University of Macedonia in Thessaloniki. Since these two departments are in the social sciences, they were chosen because students are taught similar courses. The sample comprised 36 males (14.4%) and 214 females (85.6%). Their age varied from 18 to 57 years, with a mean of 24.97 years (SD = 10.73). Regarding their academic year, 62 (24.8%) students were in their first year, 77 (30.8%) students were in their second year, 66 (26.4%) students were in their third year, and 45 (18%) students were in their fourth year and more. Academic performance in Greek universities is assessed from 1 to 10, where 5 is a grade when you pass the exam. The academic performance of our sample varied from 5.70 to 9.80, with an average grade of 8.04 (SD = .84).

### Measures

***Motivated Strategies for Learning Questionnaire (MSLQ)***

The Motivated Strategies for Learning Questionnaire (MSLQ) by Pintrich (1991) is a self-report instrument designed to assess college students’ motivational orientations and their utilization of diverse learning strategies in the context of a college course. The questionnaire consists of 33 items, and participants respond on a 7-point Likert scale ranging from 1 (absolutely disagree) to 7 (absolutely agree). Precisely, it consists of 4 items referring to intrinsic goal orientations (e.g., *In a class like this, I prefer course material that arouses my curiosity, even if it is challenging to learn.*), 4 items referring to extrinsic goal orientations (e.g., *Getting a good grade in this class is the most satisfying thing for me right now.)*, 8 items referring to self-efficacy (e.g. *I believe I will receive an excellent grade in this class.*), 5 items referring to critical thinking (e.g., *I often find myself questioning things I hear or read in this course to decide if I find them convincing.*) and 12 referring to self-regulation (e.g., *When reading for this course, I make up questions to help focus my reading.).* This instrument has already been translated in Greek ( Andreou & Metallidou, 2004). The MSLQ was used as it has been translated into numerous languages and is utilized by researchers and educators globally ( Duncan & McKeachie, 2005). The MSLQ is a valuable and reliable tool that can be adapted for various purposes for researchers, educators, and learners.

*Extrinsic and intrinsic motivation*

The questionnaire used by Pintrich (1991) measured extrinsic and intrinsic motivation. The model structure was assessed using confirmatory factor analysis, and the fit to the data was found to be good (χ ^2^(19) = 75.17, *p* < .001, CFI = .887, GFI = .994, SRMR = .060, CI 90% [0.084-0.135], RMSEA = .109). The reliability of the subscales was satisfactory: intrinsic motivation α = .754 and extrinsic motivation α = .72.

*Self-efficacy*

The model structure was assessed using confirmatory factor analysis, and the fit to the data was found to be satisfactory (χ ^2^(20) = 186.144, *p* < .001, CFI = .883, GFI = .974, SRMR = .054, CI90% [0.159-0.207], RMSEA = .182). The reliability of the subscale of self-efficacy was high, α = .92.

*Critical thinking*

The model structure was assessed using confirmatory factor analysis, and the fit to the data was found to be good (χ ^2^(5) = 44.156, *p* < .001, CFI = .936, GFI = .988, SRMR = .046, CI90% [0.131-0.227], RMSEA = .177). The reliability of the subscale of critical thinking was good, α = .86.

*Self-regulation*

The model structure was assessed using confirmatory factor analysis, and the fit to the data was found to be good (χ ^2^(27) = 62.216, *p* < .001, CFI = .954, GFI = .993, SRMR = .039, CI90% [0.049-0.096], RMSEA = .072). The reliability of the subscale of self-regulation was good α = .86.

***Achievement***

In addition, the survey included a question asking students to report their average grade across all previously completed courses. They were instructed to provide their exact average if they knew it, or to estimate it as accurately as possible (e.g. by entering an approximate value, such as 7.5). This self-reported average grade was used as a proxy for academic achievement in the statistical analysis. In line with standard practice, this measure was treated as a grade point average (GPA) on a 10-point scale, consistent with the grading system used in the institutional context of the study. Although self-reported grades may involve some estimation error, previous studies have demonstrated their strong correlation with official records, rendering them valid indicators in large-scale educational research where access to official transcripts is not feasible.

### Procedure

The recruitment process entailed the transmission of secure email invitations via Google Forms and distributing questionnaires in person at the participating universities. Before completing the primary self-report questionnaires, students had to consent and furnish demographic data. In order to ensure the protection of anonymity and compliance with the standards of confidentiality of the respondents, rigorous measures were implemented throughout the data collection process. All participants were adults who consented to participate in the study and to the publication of the findings. This procedure was conducted after obtaining approval from the Research Ethics Committee and in strict adherence to the ethical guidelines set forth by the American Psychological Association (APA) and the European Union’s General Data Protection Regulation (GDPR) regarding the management of sensitive personal data. Participants were recruited with care, and data collection was conducted over four months, from March to June 2024, strictly adhering to ethical standards and regulatory requirements.

### Data analyses

Preliminary analyses were conducted using IBM SPSS (Version 26) to explore the relationships between motivation, self-efficacy, self-regulation, critical thinking, and academic achievement. These analyses also aimed to determine the suitability of parametric tests. The procedures included descriptive statistics and intercorrelation analyses, examining means, standard deviations, skewness, kurtosis, and correlations.

Path analysis was employed using JASP ( https://jasp-stats.org/) to develop a model that explains the relationships between motivation, self-efficacy, self-regulation, critical thinking, and academic achievement. To evaluate the strength of the connections between variables, we computed standardized path coefficients (β), standard error (SE), and two-tailed p-values (considered significant at <.05). Additionally, to determine if the data aligned with our proposed model, we calculated several fit criteria, including the Chi-square test of model fit divided by degrees of freedom (χ2/df ) < 5, and additional fit indices such as the comparative fit index (CFI), goodness of fit index (GFI), Tucker–Lewis index (TLI), root-mean-square error approximation (RMSEA), and standardized root mean square residual (SRMR).

Path analysis was applied to create a model explaining the relationships between motivation, self-efficacy, critical thinking and self-regulation academic performance via JASP. To assess the strength of the paths between two variables, we calculated the standardized (β) path coefficients, the standard error (SE), and two-tailed p values (significant at <.05). In addition, to investigate whether the data fit our hypothetical model, we calculated several criteria, including values of the Chi-square test of model fit divided by degrees of freedom (χ2/df ) < 5 and supplement fit indices such as comparative fit index (CFI), goodness of fit index (GFI), Tucker–Lewis index (TLI), the root-mean-square error approximation (RMSEA), and standardized root mean square residual (SRMR). Path analysis is an appropriate methodological choice because it allows for the simultaneous analysis of multiple predictors, supporting its widespread use in motivational research and educational psychology (e.g., Zeynali et al., 2019; Kulakow, 2020). The data are available at OSF repository ( Stavropoulou et al., 2025).

## Results

### Statistical analyses

To initiate the analysis, descriptive statistics were utilized to provide a comprehensive overview of the principal measures for each variable. These statistics facilitate the comprehension of the dataset, providing insights that establish a foundational understanding. The results of this preliminary analyses are presented in Table 1, which provides detailed information on specific measures, including the mean, standard deviation, minimum, and maximum values for each variable. This table provides a valuable point of reference for interpreting the subsequent analysis.

**Table 1.** Descriptive measures for intrinsic motivation, extrinsic motivation, self-efficacy, critical thinking, self-regulation and achievement.

Descriptive statistics						
	Intrinsic motivation	Extrinsic motivation	Self efficacy	Critical thinking	Self regulation	Achievement
Valid	250	250	250	250	250	249
Missing	0	0	0	0	0	1
Mean	5.491	4.693	4.960	4.402	4.954	8.044
Std. Deviation	1.006	1.267	1.092	1.304	0.998	0.840
Skewness	-0.676	-0.419	-0.213	-0.312	-0.252	-0.338
Std. Error of Skewness	0.154	0.154	0.154	0.154	0.154	0.154
Kurtosis	0.353	-0.055	0.179	-0.166	-0.193	-0.746
Std. Error of Kurtosis	0.307	0.307	0.307	0.307	0.307	0.307
Minimum	2.000	1.000	1.125	1.000	1.900	5.700
Maximum	7.000	7.000	7.000	7.000	7.000	9.800

Path analysis was used to explore the relationships among the variables under investigation. Table 2 shows the correlation matrix of the latent variables, and Table 3 presents the regression coefficients of the path model. The correlations among the variables under investigation were low, medium, and firm positive.

**Table 2.** Correlations between intrinsic motivation, extrinsic motivation, self-efficacy, critical thinking, self-regulation, and achievement.

Variable	Intrinsic motivation	Extrinsic motivation	Self-efficacy	Critical thinking	Self regulation	Achievement
**Intrinsic motivation**	—					
**Extrinsic motivation**	0.164 *	—				
**Self-efficacy **	0.565 ***	0.194 **	—			
**Critical thinking**	0.534 ***	0.035	0.512 ***	—		
**Self-regulation **	0.561 ***	0.144 *	0.588 ***	0.540 ***	—	
**Achievement**	0.365 ***	0.076	0.427 ***	0.374 ***	0.400 ***	—

* *p* < .05.

** *p* < .01.

*** *p* < .001.

**Table 3.** Regression coefficients among the variables under investigation.

Predictor	Outcome	Estimate	Std. Error	z-value	p	Lower	Upper
Self-efficacy	Critical thinking	0.370	0.074	5.010	<.001	0.225	0.514
Intrinsic motivation	Critical thinking	0.466	0.080	5.814	<.001	0.309	0.622
	Self-efficacy	0.594	0.057	10.426	<.001	0.482	0.706
Extrinsic motivation	Self-efficacy	0.090	0.045	1.994	0.046	0.002	0.179
Critical Thinking	Self-regulation	0.185	0.044	4.196	<.001	0.099	0.271
Self-efficacy	Self-regulation	0.295	0.054	5.475	<.001	0.190	0.401
Intrinsic motivation	Self-regulation	0.248	0.059	4.162	<.001	0.131	0.364
Critical thinking	Achievement	0.143	0.044	3.297	<.001	0.058	0.229
Self-regulation	Achievement	0.235	0.057	4.126	<.001	0.123	0.346

**Figure 1.**
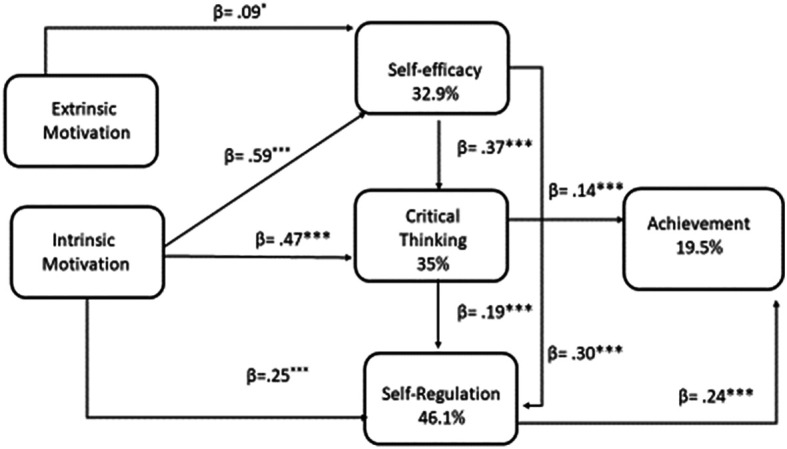
The path model describing the effects of academic factors in academic achievement.

Figure 1 shows the path model that includes direct and indirect effects, with self-efficacy, critical thinking and self-regulation acting as mediators. The model has good fit measure indices: χ ^2^ = 16.339, df = 5, p < .01; CFI = 0.998; TLI = 0.973; RMSEA = 0.095; 90% CI of RMSEA = [0.046; 0.149]; SRMR = 0.037; NNFI = 0.924; GFI = 0.999].

Figure 1 illustrates the path model that elucidates the impact of extrinsic and intrinsic motivation, self-efficacy, critical thinking, and self-regulation on academic achievement. The R ^2^ values for the dependent variable indicate that the most significant predictors are SlE (32.9%), CrT (35%), and SlR (46.1%).

As illustrated in Figure 1, the interrelationships between the variables and their respective effects are evident. The prediction of academic achievement is directly attributable to critical thinking ( *b* = .14, *p* < .001), and self-regulation (b = .24, p < .001). Self-efficacy is directly predicted by extrinsic motivation ( *b* = .09, *p* < .05) and intrinsic motivation ( *b* = .59, *p* < .001). The influence of intrinsic motivation ( *b* = .25, *p* < .01) and self-efficacy ( *b* = .37, *p* <.001) on critical thinking is direct. In conclusion, self-regulation is directly influenced by intrinsic motivation ( *b* = .25, *p* < .01), critical thinking ( *b* = .19, *p* < .001), and self-efficacy ( *b* = .30, *p* < .001).

## Discussion

The present study employed path analysis to investigate the complex relationships between extrinsic motivation, intrinsic motivation, self-efficacy, critical thinking, self-regulation, and academic achievement. By analyzing both intrinsic (internal desire to learn) and extrinsic (external rewards or pressures) motivation, as well as self-efficacy (belief in one’s ability to succeed), the study explored how these motivational constructs impact critical thinking and self-regulation—key cognitive processes that contribute to academic success. While previous research has examined some of these variables in isolation this study tries to investigate their collective influence on academic achievement through a comprehensive path model. The findings provide important information into how these interconnected factors shape overall academic achievement, offering a comprehensive understanding of their roles within the educational context. This integrated approach contributes a new perspective to the literature by demonstrating not only the direct effects of motivational constructs but also their indirect influence through self-regulatory and cognitive mechanisms. The results offer significant insights into the interactions and contributions of these variables to our comprehension of the factors influencing academic achievement.

The correlations among the latent variables exhibited varying strengths of association. Self-regulation exhibited a strong correlation with critical-thinking and self-efficacy. These findings are consistent with existing literature (e.g., Ayhan & Payan, 2023; Richardson et al., 2012; Tunçeli et al., 2022), indicating that students with higher levels of self-regulation tend to engage in more critical thinking and have greater confidence in their abilities (self-efficacy). Similarly, intrinsic motivation demonstrated strong correlations with self-efficacy and critical thinking, indicating that individuals with intrinsic motivation tend to exhibit higher self-belief and a proclivity for critical thinking. This finding is also consistent with the existing literature on the association between motivational variables and self-efficacy. For example, Greene et al. (2004), Pintrich and De Groot (1990), Stavropoulou et al. (2023), and Walker et al. (2006) have all demonstrated this relationship. Furthermore, critical thinking is associated with motivation, as evidenced by the findings of Ames and Archer (1988) and Nolen (1988). However, the present study builds on this foundation by revealing the strength and direction of these associations within a single analytical model, illustrating how critical thinking and self-regulation function as key mediators in the pathway from motivation to academic achievement.

In contrast, the correlation between extrinsic motivation and the other variables was relatively weak. For instance, the correlation between extrinsic motivation and self-regulation was relatively low, indicating that external rewards or pressures may not significantly contribute to self-regulatory behaviors compared to intrinsic factors. The weaker relationship with other constructs, such as critical thinking and academic achievement, further supports the notion that extrinsic motivation plays a less central role in fostering higher-order thinking skills and academic success than intrinsic motivation and self-efficacy. These findings align with prior research indicating that students who adopt extrinsic motivation tend to exert less effort, given their limited knowledge ( Matt & Dale, 2002). Consequently, the learning outcomes are often immediate ( Ryan & Deci, 2017, 2020). This result supports Self-Determination Theory ( Deci & Ryan, 1985), which suggests that extrinsic motivation is less effective than intrinsic motivation in promoting deep learning and sustained engagement. Learners motivated by personal interest or internalized goals are more likely to use metacognitive strategies and engage in complex thinking, whereas extrinsic rewards often lead to superficial effort. The current findings confirm that, when intrinsic motivation and self-efficacy are strong, extrinsic motivation contributes little to explaining self-regulation or critical thinking, highlighting the need to prioritize autonomous motivation in educational contexts.

The path analysis provides a more nuanced understanding of the relationships among these variables. Self-efficacy emerged as a critical predictor across multiple pathways. Not only did it significantly influence self-regulation, but it was also a strong predictor of critical thinking. The model revealed that self-efficacy significantly predicted both self-regulation and critical thinking, consistent with Zimmerman’s (2000) model of self-regulated learning. Theoretical and empirical literature (e.g., Schunk & DiBenedetto, 2020) suggests that students with strong self-efficacy beliefs are more likely to engage in complex reasoning, question assumptions, and apply evidence-based logic, which are hallmarks of critical thinking. In this sense, self-efficacy serves not only as a motivational driver but also as a cognitive enabler, empowering learners to regulate their academic behaviors and think critically. These findings suggest that self-efficacy serves as a central mechanism by which constructs such as motivation and thinking skills translate into academic success and are in line with the literature (e.g., Richardson et al., 2012; Liem et al., 2008; McGeown et al., 2012; Ng et al., 2016; Stavropoulou et al., 2023, 2024; Wolters et al., 2014). This reinforces the importance of self-efficacy as not merely a background factor but a driving force that directly impacts learners’ capacity for strategic thinking and autonomy, an insight that adds depth to existing frameworks.

About motivation, intrinsic motivation was found to have a notable effect on self-regulation and critical thinking. These findings align with those of previous studies ( Chow & Yong, 2013; Cavallo et al., 2003; Matt & Dale, 2002). In contrast, extrinsic motivation had a comparatively weaker yet still significant effect on self-efficacy. This finding is consistent with previous research indicating that extrinsic motivators have limited efficacy in promoting deep, meaningful learning ( Ryan & Deci, 2017; 2020). Specifically, extrinsic motivation in education often lead to shallow, short-term learning focused on achieving specific goals like grades, rather than mastering the material. This reliance on external rewards can undermine intrinsic motivation and reduce students’ interest in learning for personal growth. It also limits the transfer of knowledge to new contexts, fosters a fear of failure, and discourages creativity. Engagement is often temporary, lasting only as long as the reward or threat persists. Additionally, extrinsic rewards can create competitive environments, hindering collaboration and deeper learning ( Chow & Yong, 2013; Featherstone & Habgood, 2019; Liu et al., 2020; Niemiec & Ryan, 2009; Ryan & Deci, 2017, 2020). These findings collectively underscore that fostering intrinsic motivation and enhancing self-efficacy are not only beneficial but essential for developing students’ critical thinking, self-regulation, and long-term academic success.

### Predictors of academic achievement

The path model illustrates that academic achievement is directly predicted by critical thinking and self-regulation. The findings indicate that self-regulation is the most influential predictor. Self-regulation is the most influential predictor suggesting that a learner’s ability to manage their own thoughts, emotions, and behaviors plays a critical role in their success. Self-regulation includes skills such as goal setting, time management, self-monitoring, and adapting strategies to overcome obstacles that are critical to effective learning ( Meece & Painter, 2012; Pintrich, 2004). This highlights the significance of self-regulatory behaviors in academic contexts, such as goal-setting, time management, and self-monitoring. Additionally, critical thinking and self-efficacy were identified as significant contributors. These findings lend support to the notion that students who are adept at critical thinking and who exhibit a strong sense of self-efficacy are more likely to be successful in their academic pursuits. Students who excel at critical thinking can analyze information, evaluate multiple perspectives, and solve complex problems. This ability helps them engage deeply with academic material, leading to better understanding and retention. They are more likely to think independently, ask meaningful questions, and approach assignments strategically, which improves their academic performance ( García-Martín & García-Sánchez, 2018; Panadero et al., 2018; Greene & Yu, 2016; Halpern, 2013; Panadero et al., 2017). A robust sense of self-efficacy—defined as the conviction in one’s capacity to achieve success—motivates students to establish ambitious objectives and demonstrate resilience in the presence of adversity. Students who believe in their abilities are more likely to approach difficult tasks with confidence, exert the necessary effort, and persevere when faced with challenges, which directly contributes to academic success ( Alhadabi & Karpinski, 2020; Celik, 2022; Liem et al., 2008; Sheu et al., 2018). Together, critical thinking and self-efficacy create a mindset in which students are not only equipped to handle academic demands, but also motivated to push beyond their comfort zones, fostering deeper learning and long-term achievement. Nevertheless, the comparatively weaker impact of critical thinking compared to self-regulation indicates that, although indispensable, higher-order thinking skills may necessitate additional supporting factors, such as self-regulation, to translate into academic performance fully.

In conclusion, the path model identifies self-regulation and critical thinking as critical predictors of academic achievement, while self-efficacy serves as a central mediator. Although intrinsic motivation plays a significant role in these constructs, extrinsic motivation has a comparatively more minor effect. The findings underscore the necessity for educational strategies that foster self-regulation and self-efficacy, as these constructs emerge as pivotal in propelling academic success.

### Limitations

In addition to presenting findings, the current study also identifies some limitations. One limitation of the study is the use of digital questionnaires, which resulted in a convenience sample that is likely to consist of highly motivated students with strong academic performance. This may further restrict the applicability of the results to a more diverse student population. The focus on a single cultural context and the selection of participants from Educational and Psychological departments may limit the generalizability of the findings to other educational settings. Furthermore, the use of self-reported data may be susceptible to biases, such as social desirability or inaccurate self-assessment. Another limitation of the present research is that cross-sectional data do not define causal relationships. Cross-sectional studies collect data at a single point in time, providing a “snapshot” of a population or set of variables. While this type of data is useful for identifying correlations and associations between variables, it cannot determine cause-and-effect relationships.

### Suggestions for future research

Future research could benefit from several avenues to deepen our understanding of the relationships between motivation, self-efficacy, self-regulation, critical thinking, and academic achievement. Longitudinal studies are needed to track how changes in intrinsic and extrinsic motivation over time impact self-efficacy and academic success, providing insights into the long-term effects of motivational strategies. Additionally, intervention studies could focus on implementing and evaluating specific educational strategies designed to enhance these factors, assessing their effectiveness in natural classroom settings. Context-specific research is also valuable, as it can explore how cultural, socio-economic, and educational contexts influence these relationships, offering a more nuanced understanding of how different environments impact student outcomes.

Further research could explore the role of technology in supporting self-efficacy, critical thinking, and self-regulation, such as examining the impact of digital tools and gamified learning environments. Studies could also investigate how professional development programs for educators affect their ability to foster these skills in students. Additionally, exploring individual differences, such as personality traits and learning styles, and their influence on these constructs could provide insights into personalized learning approaches. Cross-disciplinary research may reveal how subject-specific strategies impact these outcomes, while mixed methods research could offer a comprehensive view by combining quantitative and qualitative data. Finally, examining the role of peer and social influences and developing improved assessment tools could further enhance our understanding and effectiveness of educational interventions.

### Theoretical and practical implications

The theoretical implications of our study extend our understanding of the interconnectedness between motivation, self-efficacy, critical thinking, and self-regulation, particularly in the context of academic achievement. Our findings support and expand existing theories that link self-efficacy with intrinsic and extrinsic motivation, demonstrating how these motivational factors serve as significant predictors of self-efficacy. This reinforces Bandura’s (1997) social cognitive theory, which posits that self-efficacy influences an individual’s cognitive processes and behaviors, including their motivation to engage in tasks. The study also adds to the body of literature that positions self-efficacy as a critical determinant of critical thinking, a fundamental cognitive process, thereby suggesting that self-efficacy not only affects how students feel about their capabilities but also how they engage in higher-order thinking skills.

Furthermore, the research highlights the role of critical thinking and self-efficacy in predicting self-regulation, thereby integrating and extending theories related to self-regulated learning. The findings suggest that self-efficacy, through its impact on critical thinking, plays a foundational role in how students plan, monitor, and adjust their learning strategies, aligning with Zimmerman’s (2000) model of self-regulated learning. Additionally, by showing that critical thinking, self-efficacy, and self-regulation collectively predict academic achievement, the study provides empirical support for theories that advocate for a holistic approach to understanding student success. These implications suggest that educational theories must consider the dynamic and reciprocal relationships between motivation, cognitive processes, and self-regulation to fully capture the complexities of academic achievement.

Our study’s results also highlight several practical implications for improving educational outcomes. First, enhancing both intrinsic and extrinsic motivation can significantly increase students’ self-efficacy, suggesting that educational programs should focus on creating engaging learning experiences and providing meaningful rewards. To further build self-efficacy and critical thinking skills, educators should incorporate activities and opportunities for students to succeed. This could include problem-based learning projects or collaborative tasks that require students to apply their knowledge in innovative ways. The key is to balance cognitive challenge with appropriate support to ensure that tasks remain within students’ zones of proximal development. The goal is not to overwhelm learners, but to design learning experiences that are sufficiently demanding to stimulate critical thinking, while also structured in a way that enables success, thereby enhancing self-efficacy.

In addition, fostering critical thinking and self-efficacy is critical to promoting effective self-regulation. Therefore, educators should design assignments that encourage students to plan, monitor, and evaluate their own progress. Tools such as self-assessment checklists, reflective journals, and peer feedback sessions can help students develop these skills. A comprehensive educational approach that addresses these components can significantly help students succeed academically by supporting their motivation, confidence, and ability to manage their own learning processes effectively. One possible area for future exploration may include examining how integrated curricular approaches uch as activities that promote cognitive engagement and self-directed learning can be implemented in ways that align with these psychological constructs. However, such educational approaches should be developed with caution and evaluated in diverse academic contexts to assess their effectiveness.

## Ethics and consent

Study-Specific Approval by the appropriate ethics committee for research involving humans: The research project was approved by the Ethics Committee of the Institute of Educational Policy of Greece (Research Section). The license number is 34/31-1-2024. The current research adheres to the Declaration of Helsinki. The date of ethical approval is 31-1-2024.

Informed consent for research involving human participants: Students completed informed consent forms for their participation in the study. The license number is 34/31-1-2024. Those who took part in this research had signed a consent form which informed them of the purpose of the research, the benefits and how it would be carried out.

## Authorship contribution statement

Stavropoulou, G.: Conceptualization, design, data collection, data analyses, writing, supervision

Daniilidou, A.: Data collection, writing, editing

Nerantzaki, K.: Data collection, writing, editing

We believe that your article type is a <include new article type>. We have gone ahead and changed your article type on your submission.

## Data Availability

**Open Science Framework:** Exploring the Interplay of Motivation, Self-Efficacy, Critical Thinking, and Self-Regulation in Predicting Academic Achievement Among University Students, DOI: https://doi.org/10.17605/OSF.IO/HM6UZ ( Stavropoulou, G et al., 2025). The project contains the following underlying data:
• Raw data as Dataset for f1000.xlsx Data are available under the terms of the Creative Commons Attribution 4.0 International license (CC-BY 4.0).
