# Supplementary material for: Exploring the Interplay of Motivation, Self-Efficacy, Critical Thinking, and Self-Regulation in Predicting Academic Achievement Among University Students
Source: F1000Res. 2025 Jul 7;14:344. Originally published 2025 Mar 28. [Version 2] doi: 10.12688/f1000research.161821.2 (PMC12345619; doi:10.12688/f1000research.161821.2)
Supplement: Peer review history

# Reviewer report: Reviewer response for version 2

**DOI:** 10.5256/f1000research.183628.r397957

**Published:** 2025-08-11

**Authors:** Felipe Sanchez Burgos

**Affiliations:** 1 Psychology, Universidad Catolica del Maule, Talca, Maule Region, Chile

From a strong literature review, the paper proposes an integration of key concepts in Higher Education learning and academic achievement: intrinsic and extrinsic motivation, critical thinking, and self-regulation. The aim here is to empirically establish how these relate between themselves, looking at how they predict academic achievement. With a fairly decent sample of HE students in Greece and through solid statistical methods, the researchers achieve their purposes in arguing for a pathway model. The research is strong and highly valuable for the field, clearly stemming from an Educational Psychology background.

 Still, from a Higher Education Research perspective, this paper could be better understood if taken in relation with this latter field. It would be fair to assume that culturally and historically Higher Education has been a privilege and luxury for society for a long time, and only recently it has been transformed by its massification and democratization, and most current by its neo liberalization and marketisation. What once was considered to be a voluntary endeavour for the privileged and capable, some would argue that now it has been increasingly believed to be a necessity (regardless of the personal/developmental or systemic/economic reasoning behind it).

 To some degree, the question this paper asks implies that students are not necessarily motivated or engaged in HE beforehand, that they may not be critical thinkers, nor that they believe to be capable, and thus constitutes a new space for academic research. Here I do not argue with these ideas, nor I challenge them; instead, I want to offer an alternative route for arguing for the value and rationale for this paper. As I see it, looking at this from the field of Educational Psychology the issue is not new per se; in contrast, it very well may be so for Higher Education Research. Of course, setting up a further and larger context would most likely change this paper substantially as much as it would support its claim. My suggestion is not to rework it but to properly extend it into the field of Higher Education as a further development for future research. I see the paper as perfectly consistent as it is, working itself from Ed Psych.

 I will elaborate on a few aspects of the paper that have not been already addressed in past reviews.

 In a general sense, the literature review does what it promises, it offers a vast and well-balanced look at the field. Still, I see two main point for further development, one internal and one external. Internally, the section would benefit from an editorial review, as it is easy to find more than a few very broad and bold statements about how the concepts relate to each other without necessarily being supported by the argumentation threads. Moreover, proofreading could be useful as (a) some sentences could be refined and (b) the text could get a more cohesive feel. Externally, I wonder if a literature review is the only section needed prior to the aim and research hypotheses section. It seems that the paper is trying to establish relationships that are not clearly taken from the literature. As the section does not refer explicitly to how the conceptual relationships are established (for example, how they are not correlational), I see that a theoretical/conceptual section could be useful. Using theory to argue for conceptual relationships could offer an avenue that empirical findings on their own do not.

 (In future projects) I would encourage the research team to  explore turning the paper on its head and start from a conceptual critique of the field and how each concept should/could relate to each other to make sense of the general learning process, for then contrasting such vision with empirical research.

 As an example of such work, I would suggest a critical review of the (intrinsic/extrinsic) motivation conceptualization. I'd question the internal consistency of such dichotomy and its value for understanding the issue. It seems that intrinsic motivation does not necessarily 'originate from within' the learner as said. In the literature review section, the argument for intrinsic motivation being influenced by a series of factors and so internalized could very well be read as it being developed or learnt. In that sense, internalization could be read from a Vygotskian perspective and so the distinction between intrinsic and extrinsic could be turned from an essential to developmental. So, the idea of an intrinsic motivation can be transformed as a more stably internalized motivation. This line of reasoning could open up the idea of a developing motivation for learning.

 This paper instead leaves the theoretical developments as hanging from empirical research without articulating internally how this may be. On the theoretical implications section, the team states that "that educational theories must consider the dynamic and reciprocal relationships between motivation, cognitive processes, and self-regulation to fully capture the complexities of academic achievement.". Still, the whole paper has been about unidirectional relationships by arguing for a path model. This is problematic because this statement reads as trying to situate itself within a holistic approach that it is in fact not taking. The conceptual relationships that have been developed here have been supported mainly by the method which sets the data into a particular order for then proving if that work. Here, the main accomplishment is that it works, but the downside is that it has been clearly underdeveloped theoretically.

 Lastly, regarding the future directions, the first paragraph seems moderately justified, yet it could use some improvement; moreover, the second paragraph reads more capricious and slightly outdated (for example, touching on learning styles which have been largely debunked).

 Overall, this paper is definitely valuable. Here, my suggestions are either cosmetic or future-oriented. Cosmetically, I suggest that the paper is reviewed to simplify and clean up its contribution. It is doing enough, yet that needs to be stated humbly and realistically. In a future oriented manner, I suggest to (i) contextualize the value of these concepts and line of research looking directly at the Higher Education research field, and (ii) theoretically address the concepts to internally articulate them, and then evaluate the benefits and hindrances of any empirical research model like the pathway one.

Is the work clearly and accurately presented and does it cite the current literature?

Yes

If applicable, is the statistical analysis and its interpretation appropriate?

Yes

Are all the source data underlying the results available to ensure full reproducibility?

Yes

Is the study design appropriate and is the work technically sound?

Yes

Are the conclusions drawn adequately supported by the results?

Yes

Are sufficient details of methods and analysis provided to allow replication by others?

Yes

Reviewer Expertise:

Higher Education; Learning & Development; Social-Constructivism; Motivation, Emotion and Learning Experiences.

I confirm that I have read this submission and believe that I have an appropriate level of expertise to confirm that it is of an acceptable scientific standard.

# Reviewer report: Reviewer response for version 2

**DOI:** 10.5256/f1000research.183628.r397256

**Published:** 2025-07-17

**Authors:** Tania Vieites

**Affiliations:** 1 Complutense University of Madrid, Madrid, Spain

I have no further comments.

Is the work clearly and accurately presented and does it cite the current literature?

Yes

If applicable, is the statistical analysis and its interpretation appropriate?

Yes

Are all the source data underlying the results available to ensure full reproducibility?

Yes

Is the study design appropriate and is the work technically sound?

Yes

Are the conclusions drawn adequately supported by the results?

Yes

Are sufficient details of methods and analysis provided to allow replication by others?

Yes

Reviewer Expertise:

Educational Psychology: self-regulation of learning, motivation, psychological well-being, academic performance, etc.

I confirm that I have read this submission and believe that I have an appropriate level of expertise to confirm that it is of an acceptable scientific standard.

# Reviewer report: Reviewer response for version 1

**DOI:** 10.5256/f1000research.177905.r379667

**Published:** 2025-05-28

**Authors:** Michelle Mingyue Gu, Lian Zhu

**Affiliations:** 1 The University of Hong Kong, Hong Kong, Hong Kong; 2 English Language Education, Education University of Hong Kong, Hongkong, Hong Kong

Journal: F1000 Research 

 Title: Exploring the interplay of motivation, self-efficacy, critical thinking, and self-regulation in predicting academic achievement among university students 

 This manuscript reports on a quantitative study on the relationship between university students’ motivation, self-efficacy, self-regulation, critical thinking, and academic achievement. A number of problems are found in the manuscript, and below are some comments that the writers need to revise: 

  

1. A stronger argument about the research gap is needed in the Introduction section. More specifically, the justification for the study needs more attention. The authors listed a number of factors affecting academic achievement and then stated that motivation is the most important one. However, why they chose to study motivation should be further explained to make the argument more convincing. Although a lack of research and possible contextual differences are good starting points, there is a need to make a case for the study based on a sound problem statement. 

 

 The authors pointed out the research concerning the relationship between motivational factors and students’ academic performance in higher education. However, there has been a lot of research conducted on motivation and academic achievement. In this regard, more literature concerning motivation and academic achievement in higher education is needed. Also, the authors may need to open the argument in a more systematic way about how this study is different from the previous ones.  

  

1. In the Literature review section, a clear definition of motivation can be put at the beginning of the section. However, the authors gave the definitions after a lot of information, which can be confusing. Also, the literature concerning motivation is too out-of-date. There have been lots of articles concerning motivation in recent years. Therefore, recent literature is needed. The researchers used intrinsic and extrinsic motivation in this paper. However, they paid more attention to intrinsic motivation rather than extrinsic motivation. It is not clear why they focused on the two motivational factors. A more nuanced statement is needed. More literature concerning extrinsic motivation is needed. Similarly, the references concerning self-efficacy are also out of date.  

 

1. The authors stated that self-regulation is highly associated with critical thinking. However, there is a lack of justification. The authors may need to provide more references to justify the importance of self-regulation in the learning process. Again, the authors pointed out the definition of the concept in the middle of the manuscript. They may put the definition at the beginning of the section to make the review clear. 

1. The participants were 250 students. However, for path analysis, the sample size was a bit small. For path analysis, the sample size should be recommended to be 10-20 times the number of parameters. However, 250 participants or more is appropriate and can be accepted. 

 

1. In terms of the measurements, the authors conducted EFA for each scale. Given that the authors obtained the developed scales in this research, only CFA is needed before conducting path analysis.  

 

1. The CFI and RMESA for self-efficacy and critical thinking are low (Hair et al., 2019). This might be because of the small sample size. Also, the measurement model can be further revised to improve the model fit for each scale before proceeding to further analysis.  

 

1. Also, the authors pointed out that students’ academic achievement was measured through their self-reported scores. However, the description of the measure was unclear. What subjects were used to examine participants’ academic achievement? Is it their GPA? Hence, a clear description of what scores were used should be further explained in the article. In addition, a serious concern is that the participants may report higher scores in data collection, which can have a huge impact on the findings.  

 

1. The literature review and discussion sections could be both strengthened and enriched by adding more recent relevant literature. Moreover, in the section of discussion, it would be necessary to provide more explanation of why the finding of the relationship between motivation, self-efficacy, critical thinking, self-regulation, and academic achievement is new to the literature. However, in this manuscript, the authors only discussed their findings in relation to previous empirical research without further discussion. This undermines the author's voice and agency. Hence, an improved and systematic discussion of how the findings are relevant to and different from and contribute to the literature.  

 

1. In the Implications section, the implications the authors outlined go beyond the data and the contexts. “For example, educators can introduce game-like elements or real-world applications to make learning more stimulating and relevant.” This is quite difficult to achieve at higher educational levels.  

 

 Additionally, some of the implications are in conflict. For instance, “To further build self-efficacy and critical thinking skills, educators should incorporate challenging activities and opportunities for students to succeed and build confidence. This could include problem-based learning projects or collaborative tasks that require students to apply their knowledge in innovative ways.”  

 Moreover, the last implication is quite strong and goes beyond the research focus. “This may involve developing a holistic curriculum that includes activities across subject areas that challenge students' cognitive skills and provide opportunities for self-regulated learning. A comprehensive educational approach that addresses these components can significantly help students succeed academically by supporting their motivation, confidence, and ability to manage their own learning processes effectively.” 

  

1. Also, the authors may need to pay attention to the grammar. Further editing is needed. For instance, “Developing critical thinking skills is paramount for students, enabling them to swiftly discern reliable information, enhance their focus on practical learning, and become attuned to real-world situations.” In addition, some of the expressions are unclear in the manuscript and should be rewritten. 

Overall, the manuscript needs substantial revision. I would not recommend the manuscript for indexing due to the research design, methods used, and its significance.

Is the work clearly and accurately presented and does it cite the current literature?

Partly

If applicable, is the statistical analysis and its interpretation appropriate?

Partly

Are all the source data underlying the results available to ensure full reproducibility?

Partly

Is the study design appropriate and is the work technically sound?

Partly

Are the conclusions drawn adequately supported by the results?

Partly

Are sufficient details of methods and analysis provided to allow replication by others?

Partly

Reviewer Expertise:

language and identity, multilingualism and mobility, family language policy

We confirm that we have read this submission and believe that we have an appropriate level of expertise to confirm that it is of an acceptable scientific standard, however we have significant reservations, as outlined above.

# Reviewer report: Reviewer response for version 1

**DOI:** 10.5256/f1000research.177905.r377235

**Published:** 2025-04-30

**Authors:** Tania Vieites

**Affiliations:** 1 Complutense University of Madrid, Madrid, Spain

**Review Summary**

 This article presents a robust and well-structured investigation into how intrinsic and extrinsic motivation, self-efficacy, critical thinking, and self-regulation influence academic achievement among university students. Drawing on a sample of 250 undergraduate students in Greece and employing path analysis, the authors provide compelling evidence for the interdependence of these variables. The research is grounded in relevant theoretical frameworks, including Self-Determination Theory and Social Cognitive Theory, and offers both theoretical and practical contributions to the field of educational psychology.

**Detailed Evaluation**

**1. Clarity and Structure**

 The manuscript is well-organized, clearly written, and easy to follow. Each section—from introduction to conclusions—is logically developed and supported with appropriate references. The use of visuals and tables enhances the reader's comprehension of complex statistical relationships.

**2. Scientific Rigor**

 The methodological approach is appropriate and thoroughly applied. The use of the MSLQ and path analysis is justified and effectively executed. The statistical reporting is clear, and the model fit indices support the conclusions drawn.

**3. Contribution to the Field**

 This study provides a valuable contribution by empirically testing the relationships between key motivational and cognitive variables in a higher education context. It addresses an important gap in the literature and offers practical implications for educational practice.

**4. Ethical Standards**

 The study was conducted with appropriate ethical oversight, and participant consent was obtained. Ethical standards are clearly met.

**5. Recommendations**

 No changes are required. The article meets the criteria for indexing in its current form.

Is the work clearly and accurately presented and does it cite the current literature?

Yes

If applicable, is the statistical analysis and its interpretation appropriate?

Yes

Are all the source data underlying the results available to ensure full reproducibility?

Yes

Is the study design appropriate and is the work technically sound?

Yes

Are the conclusions drawn adequately supported by the results?

Yes

Are sufficient details of methods and analysis provided to allow replication by others?

Yes

Reviewer Expertise:

Educational Psychology: self-regulation of learning, motivation, psychological well-being, academic performance, etc.

I confirm that I have read this submission and believe that I have an appropriate level of expertise to confirm that it is of an acceptable scientific standard.
